# A web visualization tool using T cell subsets as the predictor to evaluate COVID-19 patient's severity

**DOI:** 10.1371/journal.pone.0239695

**Published:** 2020-09-24

**Authors:** Qibin Liu, Xuemin Fang, Shinichi Tokuno, Ungil Chung, Xianxiang Chen, Xiyong Dai, Xiaoyu Liu, Feng Xu, Bing Wang, Peng Peng

**Affiliations:** 1 Wuhan Pulmonary Hospital, Wuhan Institute for Tuberculosis Control, Wuhan, Hubei Province, China; 2 Graduate School of Health Innovation, Kanagawa University of Human Services, Kawasaki, Kanagawa, Japan; Universidad Nacional de la Plata, ARGENTINA

## Abstract

Wuhan, China was the epicenter of the 2019 coronavirus outbreak. As a designated hospital for COVID-19, Wuhan Pulmonary Hospital has received over 700 COVID-19 patients. With the COVID-19 becoming a pandemic all over the world, we aim to share our epidemiological and clinical findings with the global community. We studied 340 confirmed COVID-19 patients with clear clinical outcomes from Wuhan Pulmonary Hospital, including 310 discharged cases and 30 death cases. We analyzed their demographic, epidemiological, clinical and laboratory data and implemented our findings into an interactive, free access web application to evaluate COVID-19 patient’s severity level. Our results show that baseline T cell subsets results differed significantly between the discharged cases and the death cases in Mann Whitney U test: Total T cells (p < 0.001), Helper T cells (p <0.001), Suppressor T cells (p <0.001), and TH/TSC (Helper/Suppressor ratio, p<0.001). Multivariate logistic regression model with death or discharge as the outcome resulted in the following significant predictors: age (OR 1.05, 95% CI, 1.00 to 1.10), underlying disease status (OR 3.42, 95% CI, 1.30 to 9.95), Helper T cells on the log scale (OR 0.22, 95% CI, 0.12 to 0.40), and TH/TSC on the log scale (OR 4.80, 95% CI, 2.12 to 11.86). The AUC for the logistic regression model is 0.90 (95% CI, 0.84 to 0.95), suggesting the model has a very good predictive power. Our findings suggest that while age and underlying diseases are known risk factors for poor prognosis, patients with a less damaged immune system at the time of hospitalization had higher chance of recovery. Close monitoring of the T cell subsets might provide valuable information of the patient’s condition change during the treatment process. Our web visualization application can be used as a supplementary tool for the evaluation.

## Introduction

The first case of COVID-19 infection was detected in December, 2019 in Wuhan, China. The situation quickly escalated into an epidemic all over China with tens of thousands of infections and several thousand deaths [1–3]. As of July 2, 2020, more than 10.7 million people were infected and over 515 thousands have died across the globe.

Infection of the virus weakens the host's immune system, leading to Corona Virus Disease 2019 (COVID-19). The pathophysiology of SARS-CoV-2 infection with aggressive inflammatory responses causes damage to the airways, and the disease severity depends on not only the viral infection but also the host immune response. Current estimates are that 2019-nCoV has an incubation period of 2 to 14 days, with potential asymptomatic transmission [4–7].

Hubei province is located in the central region of China and Wuhan is the capital city of Hubei. With the traffic to and from the rest parts of the country, Wuhan had become the gateway for COVID-19 epidemic in China and is the most affected area in the country [8, 9]. The situation was further worsened by the Chinese Spring Festival travel rush (from mid-January to the end of February), when the world’s largest annual human migration occurs. On January 23, a lockdown in Wuhan and other cities in Hubei province started in an effort to quarantine the epicenter. Recent statistics suggest that almost 95% of reported COVID-19 cases in China originated from Wuhan [8].

Wuhan Pulmonary Hospital was a tertiary infectious disease hospital before the outbreak, with 610 beds and ten ICU beds. When the COVID-19 epidemic started in Wuhan, it became one of the first Covid-19 designated hospitals and has received more than 700 COVID-19 patients so far. In this study we aim to provide some preliminary analysis result using several basic characteristics to evaluate the patient’s risk and progress. We have further implemented our findings into a prototype interactive web tool for more practical uses.

## Materials and methods

### Ethics approval

The study protocol was reviewed and approved by the Ethics committee of Wuhan Pulmonary Hospital (WPE 2020–8). Written informed consents were obtained from all participants before enrollment. Patient records and information were de-identified prior to the analysis.

### Process flow at the hospital

**Fig 1:**
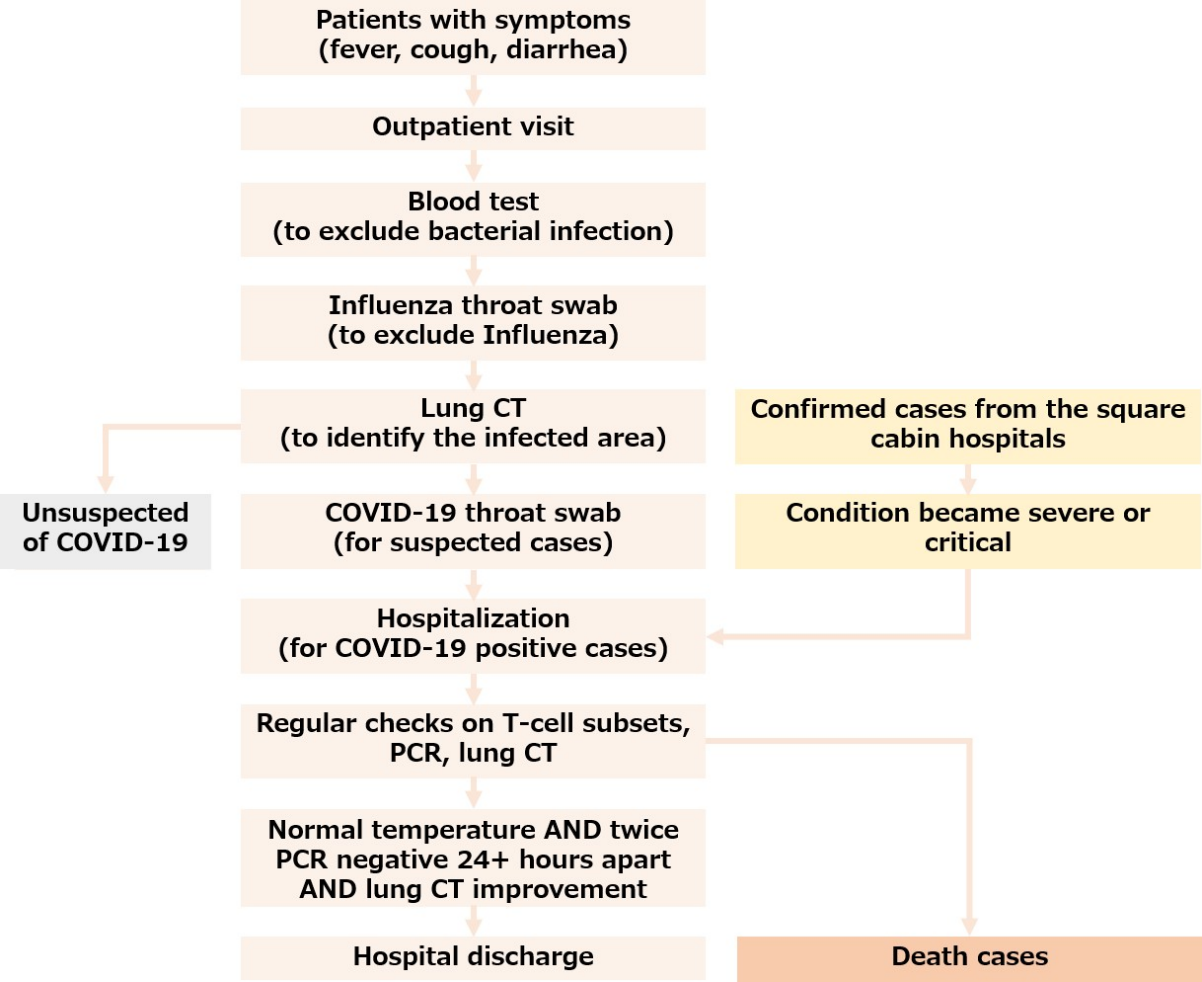
Process flow at the Wuhan Pulmonary Hospital.

Fig 1 illustrates the process flow in Wuhan Pulmonary Hospital. The patients were received from two sources: 1. Outpatient visit. People with symptoms such as fever, cough, or diarrhea made an Outpatient visit and took a blood test to exclude the possibility of bacterial infection. They then took an Influenza test to exclude the possibility of Influenza. Next a lung CT was taken to identify the infected area. Finally, a COVID-19 throat swab was taken to confirm the infection; 2. COVID-19 positive cases from the module hospital, which is generally composed of medical functional units, ward units, technical support units, etc. In February 2020, to cope with the new corona in Wuhan for the virus outbreak, the National Health Commission and related units established Wuhan Vulcan Mountain Hospital, Wuhan Thunder Mountain Hospital, and 13 Fangcang hospitals (also called as square-cabin hospitals) in Wuhan. These patients were transferred to Wuhan Pulmonary Hospital if their condition became severe or critical. Once the patient was hospitalized, regular examinations on T cell subsets, COVID-19 PCR test, and lung CT were taken place to closely monitor the patient’s progress. Most patients who were hospitalized from January 28, 2020 to March 8, 2020 received treatment according to the fifth edition of the Ministry of Health guidelines [10]: 1. Bed rest and supportive treatment; 2. Regular examination on blood routine, CRP, biochemistry, coagulation function, myocardial enzymes, lung CT; 3. Oxygen therapy, including nasal catheter oxygen, transposal high-flow oxygen therapy, ventilator; 4. Antiviral therapy, including Lopinavir/Ritonavir tablets, Arbidol Hydrochloride Tablets, etc. If the patient maintained a normal body temperature for at least three days, and tested negative with COVID-19 (including throat swab, stool, and sputum) at least twice and 24+ hours apart, as well as showing significant improvement in lung CT, the patient would be considered recovered and discharged from the hospital. The discharged patients were transported by the Chinese government using designated vehicles and they were further isolated for at least two weeks.

### Study population and data collection

From January 31 to March 8, 2020, a total of 721 patients who tested positive of COVID-19 were admitted to Wuhan Pulmonary Hospital. Among these patients, 430 completed the treatment and were discharged, 62 died, and 229 still remained in hospitalization. Excluding four patients whose direct cause of death was not COVID-19 infection, and selecting patients who had at least one T cell Subsets test available, we had a total of 340 patients in the study, including 310 discharged cases and 30 death cases. Each hospitalized patient was asked by the doctor on the day of admission to confirm whether they had any underlying disease. We reviewed laboratory test results and chest CT examinations of these 340 patients and collected all the T cell subsets tests data. If multiple T cell subsets tests were performed, we chose the earliest one as the baseline. Two researchers independently reviewed the collected data to ensure data accuracy.

### T cell subset test

For patients newly diagnosed with COVID-19 infection, 2 mL venous blood samples were obtained from each patient to measure T cell subsets. All analyses were completed within four hours of sampling in our hospital’s central lab. FACSCalibur flow cytometer (BD Biosciences, USA) was used to determine the percentages and absolute counts of the following mature human lymphocyte subsets: T lymphocytes (CD3+), Helper/inducer T lymphocytes (CD3+CD4+), and Suppressor/cytotoxic T lymphocytes (CD3+CD8+).

### Selection of analysis data

There have been a number of descriptive analyses about the epidemiological, clinical, laboratory, and radiological characteristics of the COVID-19 patients [11–15]. Little as yet known about how these characteristics can be used in guiding the practice of the healthcare providers [16, 17]. As the world face the expanding COVID-19 pandemic, with the number of infected cases increase exponentially every day, any quick and easy method of understanding the patient’s condition can be valuable. With this in mind we seek to build a statistical model with only a few strong predictive characteristics. As several other research teams pointed out, the potential risk factors of older age, high SOFA score, and d-dimer greater than 1 μg/L could help clinicians to identify patients with poor prognosis at an early stage [13]. Older patients (>65 years) with comorbidities and ARDS are at increased risk of death [18]. Additionally, during our experience of treating the patients, we have noticed that the T cell subsets are closely correlated to the patient’s progress. As the baseline T cell subsets tests were taken on the first day of the patient’s hospitalization, we observed evidence of varying degrees of decline in T cell subsets at the hospitalization. And the patient’s condition further improved or worsened with the rise or fall of the T cell subsets. We have also considered other immune indicators and inflammatory indicators, as well as the blood routine and lung CT, but they all have certain limitations. Immune indicators and inflammatory indicators such as thyrotropin and white blood cells were not as sensitive as the T cell subsets. Thyroid stimulating hormone is a hormone secreted by the pituitary gland. On the one hand, it is affected by the promotion of thyroid stimulating hormone releasing hormone secreted by the hypothalamus, and on the other hand, it is affected by the feedback inhibitory effect of thyroid hormone. Thyroid stimulating hormone is mainly responsible for regulating thyroid cells. Proliferation, thyroid blood supply, and thyroid hormone synthesis and secretion. The blood routine is not specific enough to differentiate the condition of the patients [19], and although lung CT is an accurate way to assess the patient’s condition, it cannot be repeated too often especially for those critical patients who needed oxygen and ventilator. In conclusion, we decided to focus our research on patient’s age, underlying disease status, and the T cell subsets measures: Helper T cells, Suppressor T cells, and TH/TSC (Helper T cells and Suppressor or cytotoxic T cells ratio).

## Results and discussion

### Statistical analysis results and the interactive web tool

Among the 310 discharged cases, the average age was 56.4 years (SD 14.0). 107 (34.5%) of them had underlying diseases (the comorbidities as follows: hypertension, cardiovascular disease, COPD, diabetes, chronic liver disease). The average duration of the hospitalization was 11.1 days (SD 6.94), and it took 15.1 days (SD 10.3) on average for them to reach our hospital since their first symptoms. Among the 30 death cases, the average age was 69.0 years (SD 7.87). 23 (76.7%) of them had underlying diseases. The average duration of the hospitalization was 15.1 days (SD 8.78), and it took 11.7 days (SD 8.20) on average for them to reach our hospital since their first symptoms (Table 1).

**Table 1:** Summary table of the patient characteristics and the duration of hospitalization.

	Discharged (n = 310)	Death (n = 30)	P value
**Age**			
Mean (SD)	56.4 (14.0)	69.0 (7.87)	<0.001
Median [IQR]	57.0 [46.0, 66.0]	69.0 [62.5, 73.5]	
Underlying Diseases*			
All diseases	107 (34.5%)	23 (76.7%)	<0.001
Hypertension	68 (21.9%)	16 (53.3%)	0.001
Cardiovascular Disease	19 (6.1%)	5 (16.7%)	0.048
COPD	10 (3.2%)	1 (3.3%)	>0.999
Diabetes	6 (1.9%)	2 (6.7%)	0.151
Chronic Liver Disease	6 (1.9%)	2 (6.7%)	0.151
Hyperlipidemia	4 (1.3%)	0 (0%)	>0.999
**Duration of Hospitalization (days)**			
Mean (SD)	11.1 (6.94)	15.1 (8.78)	0.024
Median [IQR]	9.00 [5.00, 14.0]	12.5 [9.25, 20.3]	
**From the First Symptom to Hospitalization (days)**			
Mean (SD)	15.1 (10.3)	11.7 (8.20)	0.039
Median [IQR]	12.0 [7.00, 20.0]	10.0 [7.00, 13.0]	
**From the First Symptom to the last day of Hospitalization(days)**			
Mean (SD)	26.3 (10.2)	26.7 (10.0)	0.806
Median [IQR]	24.5 [19.0, 34.0]	25.5 [20.3, 30.5]	

* Some patients had a combination of underlying diseases

For the discharged cases, the summary statistics for their baseline T cell subsets tests are the following: Total T cells percentage (mean 64.0%, SD 12.7%), Total T cells counts (mean 773, SD 549), Helper T cells percentage (37.2%, SD 10.6%), Helper T cells counts (mean 457, SD 342), Suppressor T cells percentage (mean 25.0%, SD 8.72%), Suppressor T cells counts (mean 297, SD 220), TH/TSC (mean 1.71, SD 0.89), and Total Lymphocyte counts (mean 1160, SD 744).

For the death cases, the summary statistics for their baseline T cell subsets tests are the following: Total T cells percentage (mean 52.7%, SD 14.2%), Total T cells counts (mean 228, SD 168), Helper T cells percentage (33.8%, SD 11.8%), Helper T cells counts (mean 139, SD 98), Suppressor T cells percentage (mean 17.3%, SD 8.72%), Suppressor T cells counts (mean 80.9, SD 97.7), TH/TSC (mean 2.41, SD 1.28), and Total Lymphocyte counts (mean 425, SD 254). Mann Whitney U test between the discharged cases and the death cases in terms of their baseline T cell subsets measures yielded significant p values: Total T cells (p = 3.2e-10), Helper T cells (p = 3.1e-09), Suppressor T cells (p = 2.1e-11), and TH/TSC (p = 0.0007) suggesting that patients with a poor prognosis had a more damaged immune system at the time of hospitalization (Table 2).

**Table 2:** Summary table of the initial T cell subsets test.

	Discharged (n = 310)	Death (n = 30)	P value (Mann Whitney U test)
**Total T Cells Percentage (%)**			
Mean (SD)	64.0 (12.7)	52.7 (14.2)	<0.001
Median [IQR]	66.2 [56.7, 73.8]	53.6 [43.7, 64.4]	
**Total T Cells Counts (cells/ul)**			
Mean (SD)	773 (549)	228 (168)	<0.001
Median [IQR]	678 [317.4, 1105.1]	170 [94.0, 339.1]	
**Helper T Cells Percentage (%)**			
Mean (SD)	37.2 (10.6)	33.8 (11.8)	0.195
Median [IQR]	37.5 [31.0, 44.5]	36.2 [24.0, 43.2]	
**Helper T Cells Counts (cells/ul)**			
Mean (SD)	457 (342)	139 (98.0)	<0.001
Median [IQR]	370 [182.8, 651.0]	115 [62.8, 195.1]	
**Suppressor T Cells Percentage (%)**			
Mean (SD)	25.0 (8.72)	17.3 (8.72)	<0.001
Median [IQR]	24.2 [19.6, 29.9]	16.3 [12.0, 20.8]	
**Suppressor T Cells Counts (cells/ul)**			
Mean (SD)	297 (220)	80.9 (97.7)	<0.001
Median [IQR]	249 [118.8, 425.1]	56.8 [26.8, 80.8]	
**Th/Tsc(TH/TSC)**			
Mean (SD)	1.71 (0.894)	2.41 (1.28)	<0.001
Median [IQR]	1.59 [1.06, 2.04]	2.25 [1.70, 2.93]	
**Total Lymphocyte Counts (cells/ul)**			
Mean (SD)	1160 (744)	425 (254)	<0.001
Median [IQR]	1020 [536.3, 1603.7]	375 [211.0, 564.2]	

**Fig 2:**
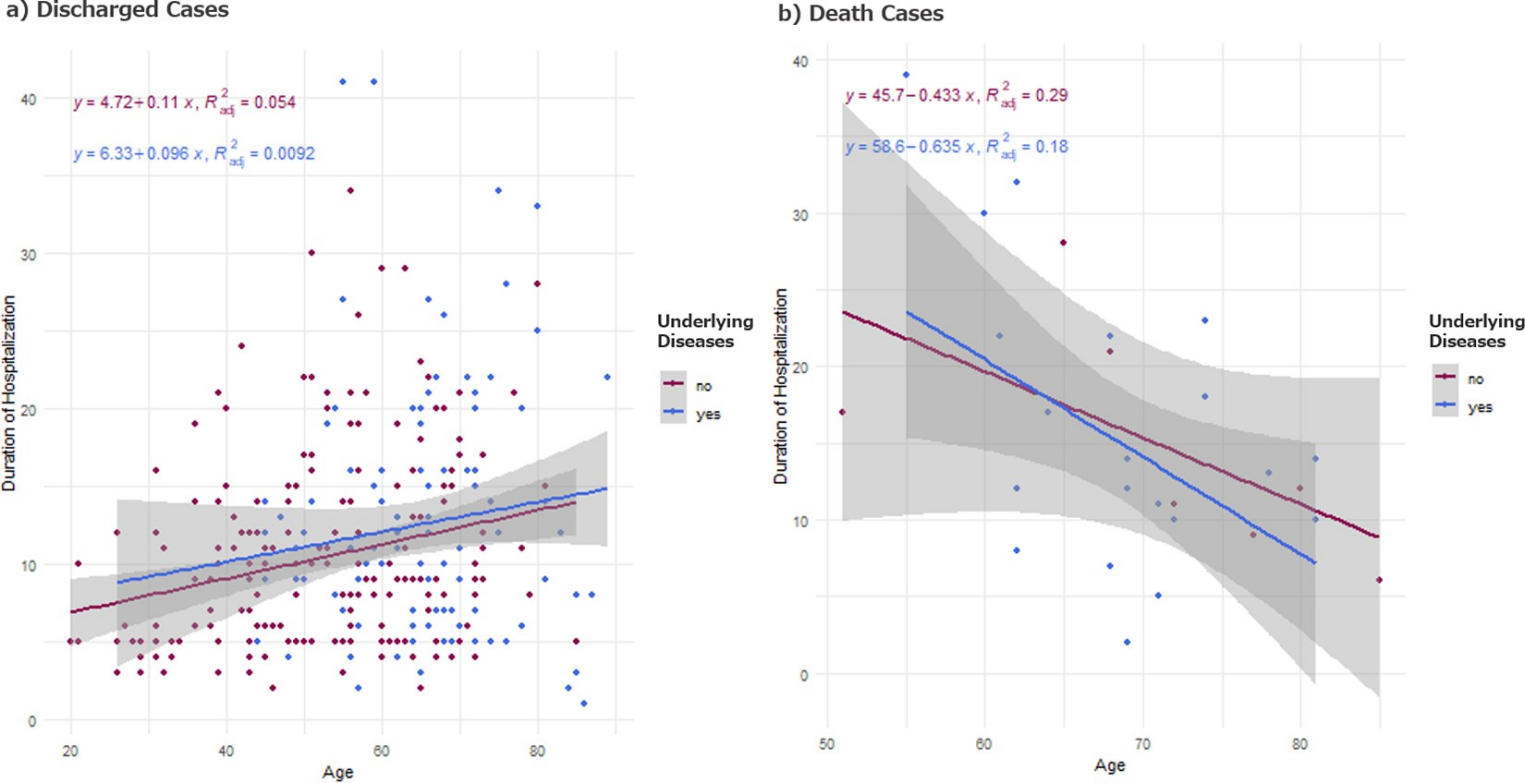
Duration of hospitalization vs age. a) Duration of the hospitalization plotted vs Age, for the discharged group. Pink dots indicate the patients with underlying medical conditions and the blue dots indicate those without. There are two observations from this chart: 1. Elderly patients tend to have underlying diseases than the younger ones; 2. The duration of the hospitalization tends to be longer among the elderly patients. The straight lines and the shaded areas show the regression line and the 95% confidence interval. The parameter estimates as well as the adjusted R squared values were also indicated in the plots. b) Duration of the hospitalization plotted vs Age, for the death group. Pink dots indicate the patients with underlying medical conditions and the blue dots indicate those without. Elderly patients die more quickly than the younger ones. The straight lines and the shaded areas show the regression line and the 95% confidence interval. The parameter estimates as well as the adjusted R squared values were also indicated in the plots.

In the scatterplots of the duration of hospitalization versus age, we can see that elderly patients tended to take longer to recover as shown by positive slopes, they also had a higher probability to have underlying diseases (Fig 2A). On the other hand, the death event occurred more quickly among elderly patients as shown by negative slopes (Fig 2B).

**Fig 3:**
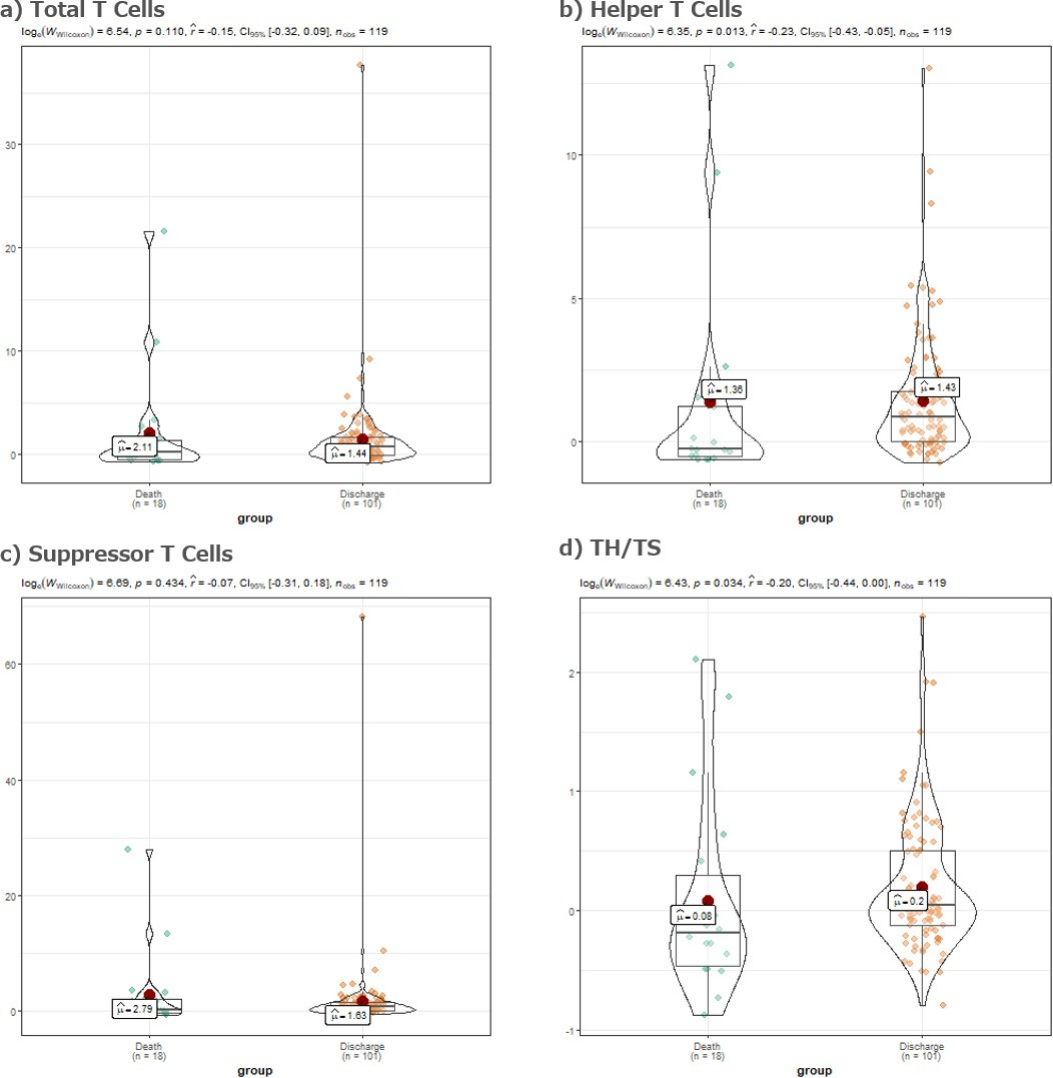
Comparison of the change rates of the T-cell Subsets between the last and first T cell subsets tests. a) Violin plot of the total T cells change rates between the last and the first T-cell Subsets tests during the hospitalization. Wilcoxon-Mann-Whitney test comparing the means of the two groups yielded a p value of 0.110. b) Violin plot of the helper T cells change rates between the last and the first T-cell Subsets tests during the hospitalization. Wilcoxon-Mann-Whitney test comparing the means of the two groups yielded a p value of 0.013. c) Violin plot of the suppressor T cells change rates between the last and the first T-cell Subsets tests during the hospitalization. Wilcoxon-Mann-Whitney test comparing the means of the two groups yielded a p value of 0.434. d) Violin plot of the total TH/TSC ratio change rates between the last and the first T-cell Subsets tests during the hospitalization. Wilcoxon-Mann-Whitney test comparing the means of the two groups yielded a p value of 0.034.

Mann Whitney U test for the change rates of the T cell Subsets between the last and first T cell subset tests for the discharged cases and the death cases yielded significant p values for Helper T cells (p = 0.013) and TH/TSC (p = 0.034) (Fig 3).

**Fig 4:**
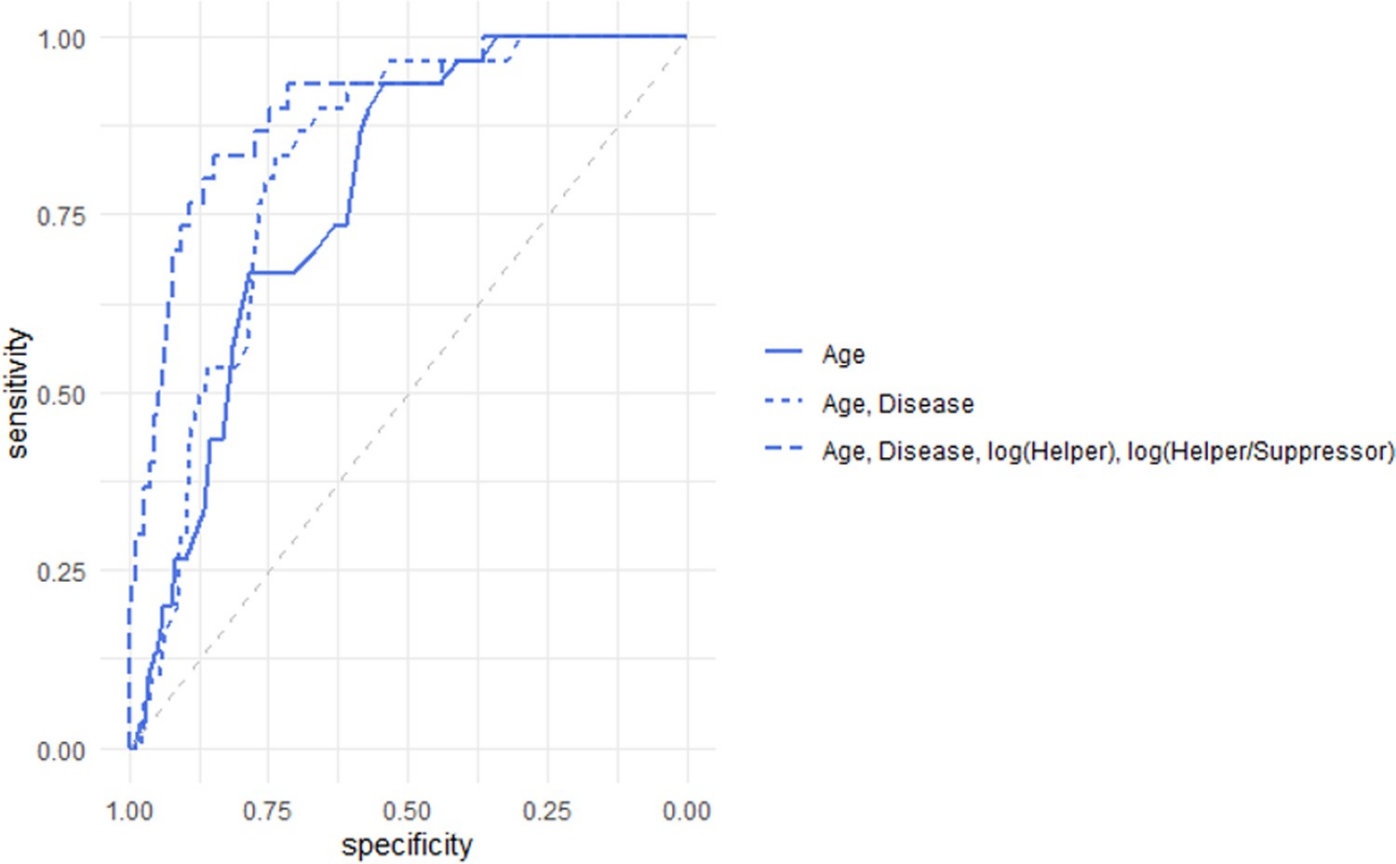
ROC Curves for three different logistic regression models. a) Model 1: logit(Probability of Death) = intercept + aAge. b)Model 2: logit(Probability of Death) = intercept + aAge + bDisease. c) Model 3: logit(Probability of Death) = intercept + aAge + bDisease + clog(Helper) + dlog(Helper/Suppressor).

We also performed a multivariate logistic regression model using age, underlying disease status, and the baseline T cell subsets test as the predictors to predict the patient outcome (death or hospital discharge). The significant predictors are age (OR 1.05, p = 0.04), underlying disease status (OR 3.42, p = 0.02), Helper T cells on the log scale (OR 0.22, p < 0.01), and TH/TSC on the log scale (OR 4.80, p < 0.01) (Table 3). The area under the ROC curve[20] for the logistic regression model is 0.90, suggesting the model has a good predictive power (Fig 4). Total T cells or Suppressor T cells, and Total Lymphocyte did not turn out to be significant predictors in our logistic regression model.

**Table 3:** Multivariate logistic regression model result using death as the outcome.

	Model 1			Model 2			Model 3		
	AUC = 0.78			AUC = 0.81			AUC = 0.9		
	95% Bootstrap CI [0.71, 0.85]			95% Bootstrap CI [0.75, 0.88]			95% Bootstrap CI [0.84, 0.95]		
	Estimate	Odds Ratio [95% CI]	P value	Estimate	Odds Ratio [95% CI]	P value	Estimate	Odds Ratio [95% CI]	P value
**Age**	0.08	1.08 [1.05, 1.13]	<0.01	0.07	1.07 [1.03, 1.12]	<0.01	0.05	1.05 [1.00, 1.10]	0.04
**Underlying Diseases**				1.25	3.49 [1.44, 9.34]	0.01	1.23	3.42 [1.30, 9.95]	0.02
**log(Helper T cells)**							-1.47	0.22 [0.12, 0.40]	<0.01
**log(TH/TSC)**							1.57	4.80 [2.12, 11.86]	<0.01

We believe by looking at some of the patient’s basic characteristics such as age and underlying diseases, together with the T cell subsets measures, could be a quick way to shed light on the patient’s prognosis, during the time of pressure and emergency. In order for our findings to be more applicable for public health workers fighting at the frontline, we have developed an interactive web data visualization tool to implement the algorithm and made it accessible for the world at the following web address: http://ec2-18-182-45-160.ap-northeast-1.compute.amazonaws.com:3838/COVID-19/.

Simply by accessing to the web address and entering a new patient’s information, the UI will show where the new patient is positioned (by a large blue dot) comparing to the reference panel of the 340 patients included in this research (S1 Fig).

This data visualization tool applied a k-means clustering method to detect the differentiation in their baseline profile patterns among all patients. K-means clustering is a type of unsupervised learning method, enabling us to find and analyze the intrinsic underlying patient subgroups without any pre-defined subgroup labels. Specifically, K-means clustering aims to partition n observations into k clusters in which each observation belongs to the cluster with the nearest mean. In real-world scenarios, clustering is a widely used technique for customer segmentation [21].

In our cluster analysis we aimed to partition n = 340 observations into k = 2 clusters, and each observation is a five-dimensional vector containing data from the following variables: age, underlying disease status, Helper T cells (log scale), Suppressor T cells (log scale), and the Helper T cells and Suppressor T cells ratio (log scale). These five variables were selected for the following reasons: 1. Age, underlying disease status, Helper T cells, and TH/TSC were significant predictors in our multivariate logistic regression model. Although Suppressor T cells was not statistically significant, it was used in the TH/TSC ratio. In fact a principal component analysis [22] showed that all of the selected five indexes carry certain proportions of independent information (S2A Fig); 2. We did not use other lab tests such as the regular blood test items in our analysis because they are less differentiative than the T cell subsets measures; and 3. We hope our interactive web tool could be utilized for quick use, therefore keeping as few input items as needed seems to be a more practical choice.

S2B Fig shows the k-means clustering result using the Wuhan Pulmonary Hospital data. After multi-dimensional data transformation, the algorithm separates the death group and the discharged group as shown in the graph. A proportion of the discharged cases had similar profiles with the death cases, which had made it difficult for the algorithm to differentiate them apart. However, by using this algorithm, it is possible to identify a large number of patients with relatively good prognosis.

We have also uploaded a dummy date set with de-identified and randomly modified patient data, as well as all the source code used for the current analysis as well as the interactive web application to: https://github.com/mindy-fang/COVID-19. All of our source code was written with the R programming language and the interactive web application was developed with the shiny package [23, 24] (Rstudio Version 1.2.1335, R version 3.6.0). Other fellow researchers can substitute the dummy data with their own data or modify the source code to make their own applications. Meanwhile we will keep updating our reference panel as we include more patients, so that the algorithm would gain more and more statistical power over time.

## Discussion

Significant reductions in T cells are very common in severe COVID-19 patients. Age-dependent deficits in T cell and B cell functions and overproduction of type 2 cytokines may cause inadequate viral replication control and longer pro-inflammatory responses, which may lead to poor prognoses [25]. Lymphopenia is a prominent part of SARS-CoV infection and lymphocyte counts may be useful in predicting the severity and clinical outcomes [26], as 2019-nCoV was called earlier Severe Acute Respiratory Syndrome Coronavirus 2 (SARS-CoV-20) [27]. The level and the speed of T cell recovery are important factors of assessing disease prognoses and guiding early intervention in critically ill patients. The study purposes includes:1, we have identified that older age, underlying diseases, and lower T cell counts may be risk factors for poor clinical outcomes in COVID-10 positive patients; 2, We have also implemented an interactive web tool to visualize a new patient’s risk using these risk factors. 3, Another important significance of our tool is the monitoring of patient’s progress in real time during the treatment. For example, if we take repeated measures of the patient’s T cell subsets test and visualize the patient’s relative position with our app, the patient’s relative position as shown in the app gradually shifting towards the red centroid (poor outcome) or the green centroid (good outcome) may provide insight on the patient’s progress (S1 Fig). This feature can help clinicians to evaluate the condition of a new patient and to ring alarm for critically ill patients.

It should be noted that our intension is not for the web tool to be considered as a 100% gold standard for determining the final clinical outcome of the patients. The actual final prognosis of a patient depends on many other factors, including the time of treatment initiation, adherence to treatment, and extent of treatment. The visualization result simply suggests whether the new patient is more likely to recover or to have adverse outcomes.

Our algorithm has the following limitations: It may not predict accurately for younger patients, or patients with no symptoms, since our training data contains relatively senior and severe patients; Secondly, the model was built based on a sample size of 340, which is not a large number. When we collected our data, 229 patients still remained in hospitalization, so we excluded them for lack of definite clinical outcome. As the early phase of the COVID-19 outbreak, we did not fully realize the importance of the T cell subgroup test so 152 patients did not have the test data. But we will keep updating our web application as we collect more data from the remaining 200+ patients in our hospital; Thirdly, our understanding about the disease is still preliminary. We expect more findings about the underlying mechanism of the disease to be published by other researchers, including how the virus infects the body, how the body reacts to the infection, how the infection changes the vital status, and so on. We are also doing an exploratory research using other biomarkers such as interleukin-6, and other surrogate clinical outcomes such as oxygen-free survival time.

In addition, the reference panel used in our analysis were infected population in Wuhan, China. Although there was no clear evidence that the underlying mechanism of the T cell depletion under the COVID-19 infection is similar or different across ethnicity groups, it is only natural to assume that the baseline values and the degree of T cell depletion would be slightly different. In fact, there had already been evidences that the T-lymphocytes, B-lymphocytes, and NK cells differ among people in different regions [28–33]. We encourage researchers around the world to download the source code and customize it with their own data, adding more clinical characteristics and biomarkers as they accumulate more experience and knowledge with patients from their own hospitals or regions.

## Conclusions

Our study has found that elderly patients with underlying diseases, lower T cell subsets counts may have a high risk of poor prognosis for COVID-19 infection. Our web visualization tool was used at Wuhan Pulmonary Hospital to serve as a quick assessment for a patient’s risk at the time of examination and to keep a close monitoring of the patient’s condition change in a timely manner. However, in order for the tool to be practically useful for other research teams or hospitals, further development and customization of the web tool is warranted by adding more clinical characteristics and biomarkers.

## Supporting information

S1 Fig Screen shots of the interactive web tool. a) A 60 year old patient with underlying diseases, displayed by the large blue square, showing a critical condition with Helper T cell counts of 100 and Suppressor T cell counts of 100. b) The same patient showing improved condition, with Helper T cell counts increased to 600 and Suppressor T cell counts increased to 500.
(TIF)

S2 Fig Principal component analysis and cluster analysis. a) The three selected T-cell subsets indexes and age, underlying disease indicator were plotted against their first two principal components, which carries 45.42% and 23.56% of the total information respectively. The dots in the background represent each patient. There was very little overlap among the six variables suggesting them carrying exclusive information. b) Multi-dimensional transformation of the above five variables for the 340 patients in our study, so that the underlying patterns can be recognized to the maximum extent. A proportion of the discharged cases had similar T-cell subsets profiles with the death cases which made it difficult to differentiate between them.
(TIF)
