# Supplementary figures and images for: A web visualization tool using T cell subsets as the predictor to evaluate COVID-19 patient's severity
Source: PLoS One. 2020 Sep 24;15(9):e0239695. doi: 10.1371/journal.pone.0239695 (PMC7514096; doi:10.1371/journal.pone.0239695)

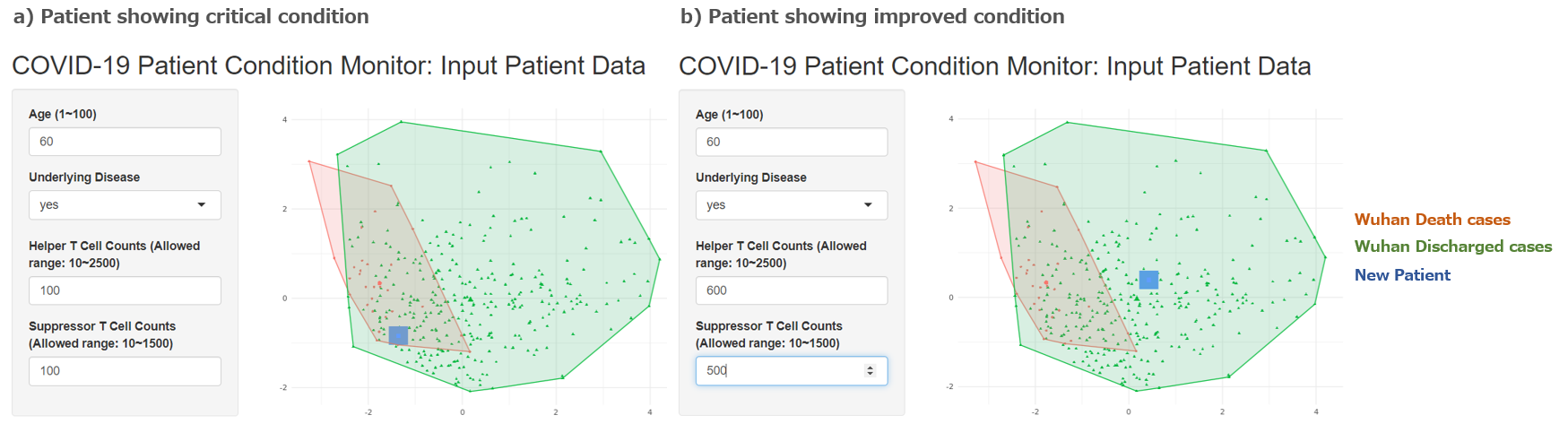

Supplement: S1 Fig — a) A 60 year old patient with underlying diseases, displayed by the large blue square, showing a critical condition with Helper T cell counts of 100 and Suppressor T cell counts of 100. b) The same patient showing improved condition, with Helper T cell counts increased to 600 and Suppressor T cell counts increased to 500. (TIF) [file pone.0239695.s001.tif]

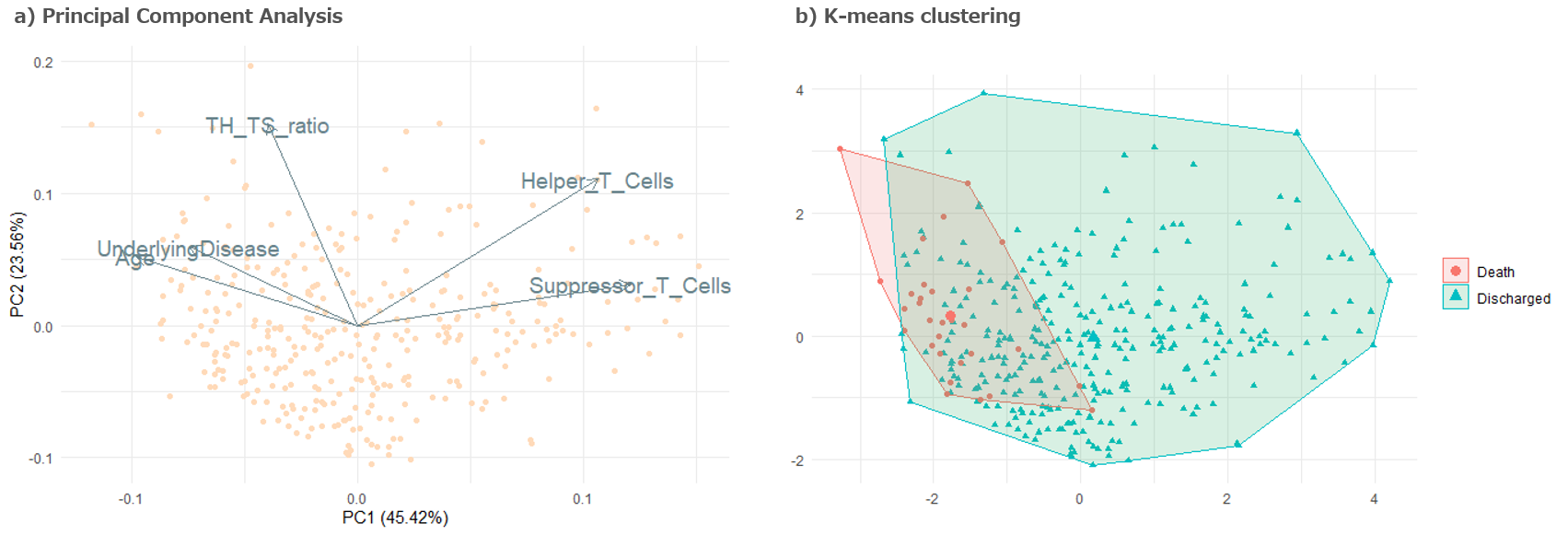

Supplement: S2 Fig — a) The three selected T-cell subsets indexes and age, underlying disease indicator were plotted against their first two principal components, which carries 45.42% and 23.56% of the total information respectively. The dots in the background represent each patient. There was very little overlap among the six variables suggesting them carrying exclusive information. b) Multi-dimensional transformation of the above five variables for the 340 patients in our study, so that the underlying patterns can be recognized to the maximum extent. A proportion of the discharged cases had similar T-cell subsets profiles with the death cases which made it difficult to differentiate between them. (TIF) [file pone.0239695.s002.tif]
